# Case Report: Successful therapy with all-*trans* retinoic acid combined with chemotherapy followed by hematopoietic stem cell transplantation for acute promyelocytic leukemia carrying the *BCOR-RARA* fusion gene

**DOI:** 10.3389/fonc.2022.1013046

**Published:** 2022-09-23

**Authors:** Li Chen, Hongming Zhu, Yongmei Zhu, Wen Jin, Fangyi Dong, Jianfeng Li, Jiong Hu, Qiusheng Chen, Kankan Wang, Junmin Li

**Affiliations:** Shanghai Institute of Hematology, State Key Laboratory of Medical Genomics, National Research Center for Translational Medicine at Shanghai, Ruijin Hospital Affiliated to Shanghai Jiao Tong University School of Medicine, Shanghai, China

**Keywords:** acute promyelocytic leukemia, variant, BCOR-RARA, all-trans retinoic acid, allogeneic hematopoietic stem cell transplantation

## Abstract

Acute promyelocytic leukemia (APL) is characterized by the balanced translocation of chromosomes 15 and 17, resulting in the formation of *PML-RARA* fusion gene. More than 98% of APL have *PML-RARA* fusion, and less than 2% have other types of *RARA* gene partners, which named variant APL (vAPL). In the present study, we reported a vAPL with *BCOR-RARA*, which was the third case of *BCOR-RARA* APL published. The patient achieved complete remission (CR) with all-*trans* retinoic acid (ATRA) monotherapy, and molecular CR with ATRA plus standard chemotherapy. After that, he underwent allogeneic hematopoietic stem cell transplantation (allo-HSCT) and ATRA maintenance and maintained a molecular CR status. This case provided valuable insights into the accurate identification of vAPL. Moreover, ATRA combined with chemotherapy followed by allo-HSCT was suggested as an optimal choice for those vAPL patients who had a high risk of relapse.

## Introduction

Acute promyelocytic leukemia (APL) is characterized by a clonal expansion of abnormal promyelocytes in bone marrow. The majority of the patients manifest the t(15;17) translocation forming the *PML-RARA* fusion gene, regarded as classical APL. In a small number of APL cases, *RARA* is fused with an alternative gene partner, such as *ZBTB16, NPM1*, and *STAT5B*, termed as variant APL (vAPL). To date, at least 16 *RARA* variant gene partners have been identified, most of which have been reported as rare cases or even a single case, in addition to *ZBTB16* (1–3). Most patients with vAPL are insensitive to arsenic trioxide (ATO) and/or all-*trans* retinoic acid (ATRA), and their prognosis is far worse than classical APL (4, 5). To our knowledge, 2 cases of APL with *BCOR-RARA* have been published, and here we report the third case.

## Case presentation

**Figure 1:**
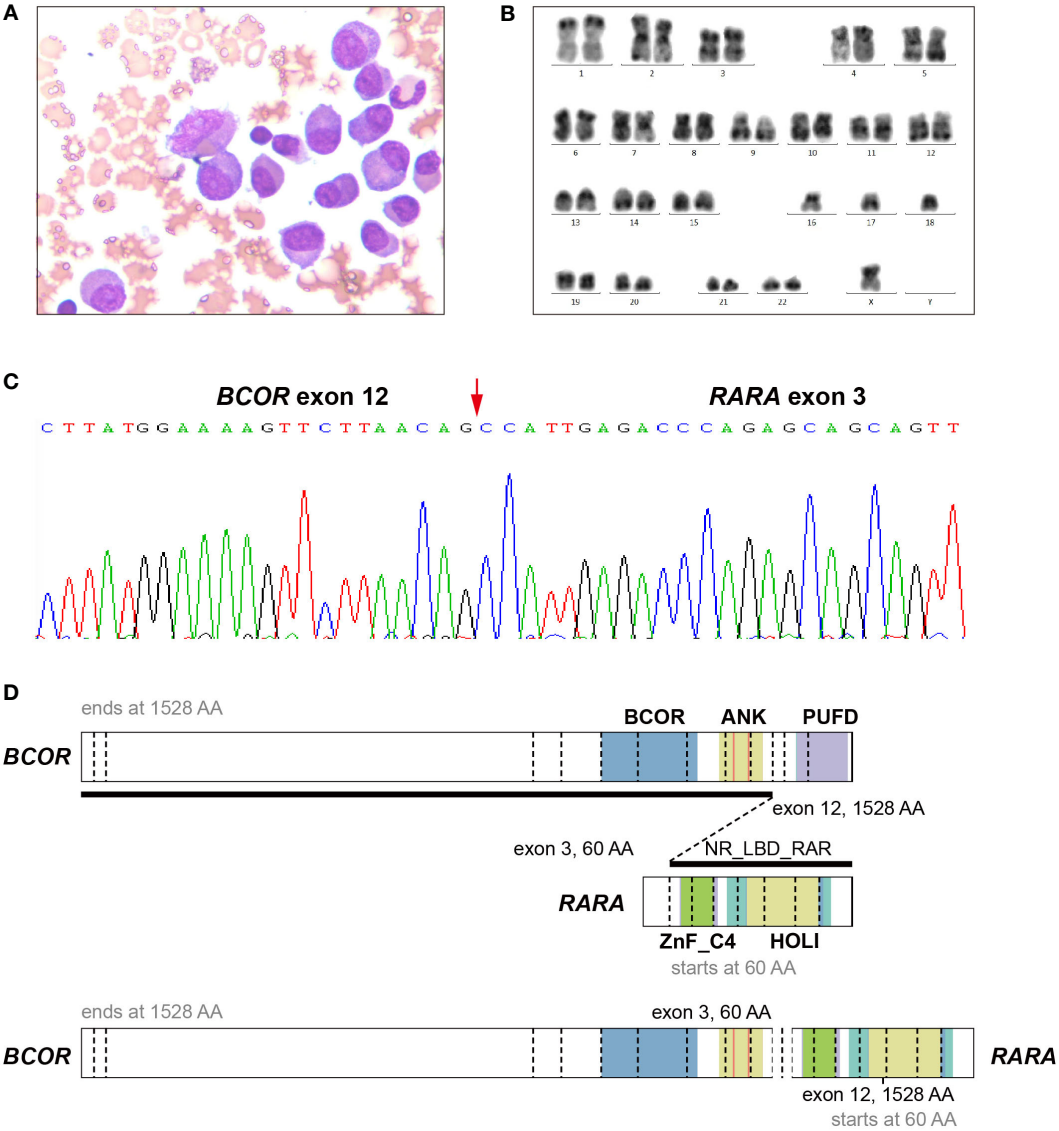
Morphology, cytogenetic and Molecular analysis of a bone marrow sample of the APL patient with BCOR-RARA. **(A)** May-Giemsa staining. Original magnification × 400. **(B)** Karyotype analysis. 42, X, -Y, -15, -16, -18 was detected in the patient. **(C)** BCOR-RARA fusion sequence at the junction site. An in-frame BCOR-RARA transcript is shown with corresponding exon numbers. The junction is indicated by a red arrowhead. **(D)** Schematic representation of BCOR, RARA, and the BCOR-RARA fusion protein. BCOR-RARA protein retains both BCOR and ANK of BCOR and ZnF_C4 and NR_LBD of RARA. ANK, ankyrin repeats; PUFD, PCGF Ub-like fold discriminator of BCOR; NR_LBD_RAR, the ligand binding domain (LBD) of retinoic acid receptor (RAR); ZnF_C4, c4 zinc finger in nuclear hormone receptors; HOLI, Ligand binding domain of hormone receptors.

**Figure 2:**
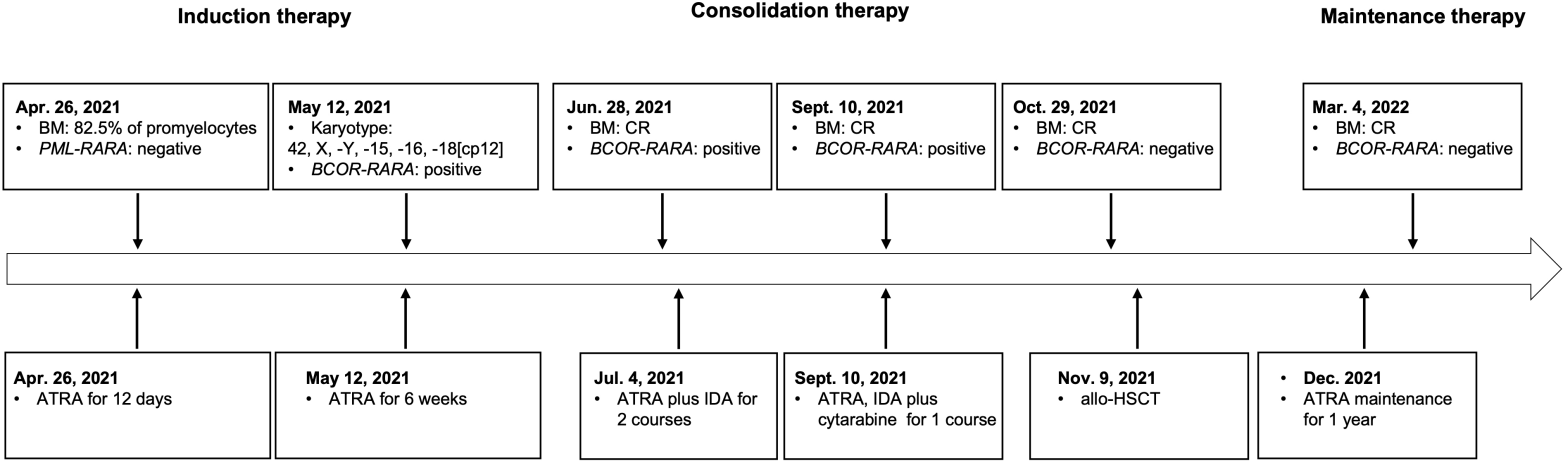
Timeline with the main events of the clinical episode for the patient.

The patient was a 47-year-old man who was admitted to a local hospital (the First Affiliated Hospital of Nanchang University) in April 2021. He suffered from dizziness, fatigue and exertional dyspnea for 2 weeks. A full blood count showed a white blood cell count of 10.05 × 10^9^/L, a hemoglobin level of 53 g/L, and a platelet count of 108 × 10^9^/L. The prothrombin time and activated partial thromboplastin time were within the normal range. Fibrinogen and D-dimer levels were 4.43 g/L (reference range 2-4 g/L) and 5.43 mg/L (reference < 0.5 mg/L), respectively. The morphological analysis of bone marrow (BM) showed extreme hyperplasia with 82.5% of hypergranular promyelocytes, and no Auer rod was found in his promyelocytes (**Figure 1A**). Flow cytometry revealed that the abnormal cells expressed CD13, CD33, CD117, CD38, CD56, but lacked the expression of CD34, CD15, CD14 and HLA-DR. The patient was proposed to be diagnosed as APL and was treated with ATRA. However, both the reverse transcription-polymerase chain reaction (RT-PCR) and fluorescence *in situ* hybridization (FISH) failed to provide any evidence of the *PML-RARA* fusion transcript in his BM. As a result, he suspended ATRA treatment and came to Ruijin Hospital affiliated to Shanghai Jiao Tong University School of Medicine. The karyotype analysis indicated 42, X, -Y, -15, -16, -18[cp12] (**Figure 1B**). Results of a multiplex RT-PCR panel covering 49 fusion genes commonly found in myeloid leukemia, including *PML-RARA*, *PLZF-RARA*, *NPM1-RARA*, *STAT5b-RARA*, *NuMA1-RARA*, *PRKARIA-RARA* and *FIPIL1-RARA*, were negative. Then, we performed RNA sequencing (RNA-Seq) on BM samples from our patient, and found the existence of the *BCOR-RARA* fusion transcript, in which exon 12 of *BCOR* was fused with exon 3 of *RARA* (**Figures 1C, D**). Furthermore, we performed targeted next-generation sequencing (NGS) covering 100 genes reportedly mutated in myeloid leukemia. We found mutations of *NRAS*, *KRAS*, *FLT3*-ITD, *FLT3*-TKD in his blasts. Hence, the patient was diagnosed with vAPL and continued induction therapy with ATRA. At the same time, hydroxyurea was used to control leukocytes and dexamethasone was used to prevent differentiation syndrome. Four weeks after hospitalization (June 9, 2021), the BM smear showed 10% of promyelocytes, 20.5% of abnormal neutrophilic myelocytes with nucleocytoplasmic imbalance, 3% of metamyelocytes and 4.5% of neutrophilic stab granulocytes. The patient was then discharged from our hospital and continued to take ATRA. On June 28, 2021, the BM smear showed complete remission (CR), and minimal residual disease (MRD) measured by flow cytometry was less than 0.01%, but *BCOR-RARA* measured by RT-PCR was still positive. The patient was then treated with ATRA plus idarubicin (IDA) (ATRA 20 mg twice daily, days 1-14, IDA 8 mg/m^2^ days 1-3) for 2 courses. The third consolidation course of chemotherapy plus ATRA after 2 courses of ATRA plus IDA had been intensified by adding cytarabine (ATRA 20 mg twice daily, days 1-14, IDA 8 mg/m^2^ days 1-2, cytarabine 100 mg/m^2^ days 1-5) because of the failure of molecular remission, and finally his *BCOR-RARA* turned negative. Then, he underwent related haploidentical allogeneic hematopoietic stem cell transplantation (allo-HSCT) on November 9, 2021. The conditioning regimen included fludarabine 30 mg/m^2^/d from day -6 to day -2, busulfan 3.2 mg/kg/d from day -6 to day -5 and melphalan 70 mg/m^2^/d from day -3 to day -2. The graft-versus-host disease (GVHD) prophylaxis consisted of cyclophosphamide 50 mg/kg on day 3 and day 4 followed by tacrolimus 0.05 mg/kg divided into 2 doses per day from day 5 (6). However, a single dose of 2.5 mg/kg anti-thymoglobulin (ATG) after neutrophil engraftment was omitted because of pneumorrhagia on day 12. After that he received ATRA maintenance for 1 year (ATRA 20 mg twice daily, days 1-14, every month). Until now, he has maintained a molecular CR status for more than 9 months after HSCT. **Figure 2** shows the detailed events of the clinical episode for the patient.

## Discussion

APL variants account for 2% of APL, and the expansion in the detection of *RARA* partners might be attributed to the advancements in transcriptome sequencing. These variants are different from the classical APL in many ways, including the clinical presentations, morphological and cytochemical characteristics, and immunophenotyping, which could delay the final diagnosis of vAPL. vAPL often poses a management challenge as there are no defined guidelines. The outcome seems to be suboptimal in a number of cases with poor response to ATRA and ATO, such as *ZBTB16-RARA*. Still, fusions, such as *NPM1-RARA* and *FIP1L1-RARA*, are sensitive to ATRA, and fusions, such as *TTMV-RARA*, are sensitive to both ATRA and ATO combination therapy (4, 7). Generally, these variants should be treated with a combination of ATRA and chemotherapy (anthracyclines), with the possible use of AML protocols in known resistant variants (4, 8, 9).

APL with *BCOR-RARA* is quite rare among vAPL. Our results showed that this kind of vAPL could also be accompanied by common gene mutations in classical APL or other acute myeloid leukemia (AML), which may also be one of the reasons for high risk of relapse. Although these patients could achieve remission after ATRA combined with traditional chemotherapy, they could barely be cured.

BCOR is a transcriptional corepressor by the interaction of the proto-oncoprotein, BCL6 and plays critical roles in myeloid differentiation (8). The incidence of *BCOR* mutations is about 3.8% to 5.0% in adult *de novo* AML (10), and loss-of-function mutations in *BCOR* serve as an independent risk factor for poor outcomes of AML (11). The subcellular localization of BCOR-RARA is distinct with wild-type BCOR, likely destroying the function of wild-type BCOR (10). The difference in ATRA sensitivity might be related to variation in corepressors (12). *BCOR-RARA*, similar to *PML-RARA*, has a high affinity for corepressor molecules, thereby requiring high levels of ATRA to induce release of the corepressor complex and allow transcription and differentiation to proceed. Therefore, patients with *BCOR-RARA* can achieve CR with the same therapy as patients of classical APL (13). Our patient also achieved hematological remission with ATRA monotherapy.

The morphology of blast cells of our patient did not show the rectangular and round cytoplasmic inclusion bodies as reported by Yamamoto et al., but the immunophenotyping by flow cytometry was similar to that of the other 2 patients, with expression of CD13, CD33 and lack of HLA-DR, which may be a feature of APL with *BCOR-RARA*. Besides, our patient and the one described by Yamamoto et al. had a strong expression of CD56, while the other one did not (**Table 1**). It has been reported that CD56 is frequently expressed in some variant APL forms (13, 15). In addition, our patient had mutated *NRAS*, *KRAS*, *FLT3-*ITD and *FLT3*-TKD. *BCOR*-mutated AML patients usually exhibit a high rate of *N-RAS* and *K-RAS* mutations (36.8%). Moreover, *BCOR*-mutated cases show a lower remission rate, overall survival and relapse-free survival, as compared with cases of wild-type. HSCT seems to abrogate the adverse prognostic impact of *BCOR* mutations (10). Although *BCOR* mutations were undetected in our patient, the fusion of *BCOR* and *RARA* may affect the function of *BCOR* gene by altering the subcellular location of wild-type BCOR. *FLT3*-ITD mutation is a poor prognostic factor not only for AML, but also for APL in the era of ATRA combined with chemotherapy (16). Recently, we analyzed the genomics and transcriptomics in 348 newly diagnosed APL patients and found that *NRAS* mutation was an independent adverse prognostic factor for APL (17). We did not find t(X;17)(p11;q12) chromosomal translocation by conventional chromosome banding test, but complex karyotype with 42, X, -Y, -15, -16, -18. These genomic and chromosomal abnormalities further supported the indication to allogeneic transplantation for this patient.

**Table 1:** Comparison of clinical features of the three cases of acute promyelocytic leukemia with *BCOR-RARA* fusion.

Author (year) (Refs.)	Present study	Yamamoto Y (2010) (12)	Satoshi Ichikawa (2015) (14)
Age	47	45	71
Gender	Male	Male	Male
Lab test			
WBC (×10^9/L)	10.05	25.3	leukocytosis
Hb (g/L)	53	121	anemia
PLT (×10^9/L)	108	116	normal
DIC	APTT, PT normal; Fg 4.43 g/L; DD 5.43 mg/L	INR 1.58, APTT 33.7s, Fg 52 mg/dL, FDP 50.6 mg/L	trivial
BM morphology	hypergranular promyelocytes, no Auer rod	peculiar rectangular and round cytoplasmic inclusion bodies in APL cells	not characteristic of APL, few Auer bodies, no faggot cells
Karyotype	42, X, -Y, -15, -16, -18	t(X;17)(p11;q12)	45, - Y, t(X;17)(p11.4;q21)
Flow cytometry	CD13+, CD33+, CD117+, CD38+, CD56+, CD34-, CD15-, CD14-, HLA-DR-	CD13+, CD33+, CD56+, HLA-DR-	CD13+, CD33+, HLA-DR-, CD34-, CD56-, and CD11c-
NGS	mutations of *NRAS, KRAS*, *FLT3-ITD*, *FLT3-TKD*	NA	NA
Treatment			
Induction treatment	ATRA only	ATRA + IDA + cytarabine	IDA + cytarabine
Achieve CR	Yes	Yes	Yes
Consolidation treatment	ATRA + IDA 2 courses, ATRA + IDA + cytarabine 1 course	MTN + cytarabine, DNR + cytarabine, IDA + cytarabine	ATRA + chemotherapy 3 courses
Transplantation	allogeneic HSCT	cord-blood transplantation	No
Outcome	mCR	CR3	mCR maintained for 1 year
Survival (months)	> 15	> 41	> 12

WBC, white blood cell; Hb, Hemoglobin; PLT, platelet; DIC, disseminated intravascular coagulation; BM, bone marrow; NGS, next-generation sequencing; CR, complete remission; APTT, activated partial thromboplastin time; PT, prothrombin time; Fg, Fibrinogen; DD, D-dimer; INR, international normalized ratio; FDP, fibrin/fibrinogen degradation products; APL, acute promyelocytic leukemia; NA, not available; ATRA, all-trans retinoic acid; IDA, idarubicin; MTN, mitoxantrone; DNR, daunorubicin; HSCT, hematopoietic stem cell transplantation; mCR, molecule complete remission.

Our previous studies have shown that the addition of targeted drugs could reduce the intensity of chemotherapeutics, thereby alleviating severe myelosuppression caused by chemotherapy. Therefore, the patient was applied with ATRA plus IDA as consolidation for 2 courses, the same as the scheme of non-ATO group for low- and intermediate-risk APL patients in the APL2012 study (NCT01987297) (18). Nevertheless, *BCOR-RARA* was still positive, suggesting that ATRA plus anthracycline was not enough to clear MRD in such cases. We added cytarabine to the ATRA plus IDA regime as the third consolidation, and the *BCOR-RARA* fusion gene of the patient turned negative eventually. Therefore, it is suggested that ATRA combined with traditional AML chemotherapy may be a better option for APL with *BCOR-RARA*.

Although patients with *BCOR-RARA* could achieve promising short-term efficacy by ATRA plus chemotherapy, relapse still remains a major concern for patients. All the 3 patients received ATRA-chemotherapy-based treatment. The first case of APL with *BCOR-RARA* was reported with 2 episodes of relapse, and achieved the third remission by intensive chemotherapy plus cord-blood transplantation. The second case maintained molecular remission by intensive chemotherapy plus ATRA for 1 year so far, and long-term follow-up was anticipated (12, 14). The detailed treatment and outcome of the 3 patients are shown in **Table 1**. Transplantation was rarely used as a modality for vAPL, given the short-term follow-up and small number of the cases, but the benefit has been discussed in some variants with the high relapse rate, like S*TAT5B-RARA* (9) and very young cases, like *TTMV-RARA* (7). Our patient had even stronger indications for transplantation due to complicated chromosome karyotype and poorly prognostic mutated genes. Besides, he continued taking ATRA as maintenance after transplantation since this setting of APL was sensitive to ATRA.

Future considerations for vAPL should include 1) the improvement of fast detection for these variants, not only the popularization of genetic sequencing, but also the use of artificial intelligence tools for deep learning of morphologic features just as in classical APL (2, 19, 20) the investigation of more therapies that might provide a better outcome, including hypomethylating agents, Bcl-2 inhibitors and hematopoietic cell transplantation.

In summary, our detailed analysis of this rare case of vAPL has showed that ATRA combined with traditional chemotherapy could bring good short-term outcome to APL patients with *BCOR-RARA*. Furthermore, allo-HSCT was administered as an optimal choice to cure the patient considering a high risk of relapse.

## Data availability statement

The datasets presented in this article are not readily available because of ethical/privacy restrictions. Requests to access the datasets should be directed to the corresponding author.

## Ethics statement

The studies involving human participants were reviewed and approved by the Ethics Committee of Ruijin Hospital in Shanghai. The patients/participants provided their written informed consent to participate in this study.

## Author contributions

JuL designed the study. LC, HZ and YZ wrote the paper. KW revised the manuscript. FD and QC followed the patient. JH performed allogeneic hematopoietic stem cell transplantation for the patient. WJ and JiL performed the molecular studies. All authors contributed to the article and approved the submitted version.

## Funding

National Natural Science Foundation of China (No. 81800141, 81890994, 81770144 and 81870110). Science and Technology Commission of Shanghai Municipality (No. 19DZ1910702).

## Conflict of interest

The authors declare that the research was conducted in the absence of any commercial or financial relationships that could be construed as a potential conflict of interest.

## Publisher’s note

All claims expressed in this article are solely those of the authors and do not necessarily represent those of their affiliated organizations, or those of the publisher, the editors and the reviewers. Any product that may be evaluated in this article, or claim that may be made by its manufacturer, is not guaranteed or endorsed by the publisher.
